# Genomic Characterization of Carbapenem-Non-susceptible *Pseudomonas aeruginosa* Clinical Isolates From Saudi Arabia Revealed a Global Dissemination of GES-5-Producing ST235 and VIM-2-Producing ST233 Sub-Lineages

**DOI:** 10.3389/fmicb.2021.765113

**Published:** 2022-01-06

**Authors:** Michel Doumith, Sarah Alhassinah, Abdulrahman Alswaji, Maha Alzayer, Essa Alrashidi, Liliane Okdah, Sameera Aljohani, NGHA AMR Surveillance Group, Majed F. Alghoribi, Hanan H. Balkhy, Majed F. Alghoribi

**Affiliations:** ^1^Infectious Diseases Research Department, King Abdullah International Medical Research Center, Riyadh, Saudi Arabia; ^2^King Saud bin Abdulaziz University for Health Sciences, Riyadh, Saudi Arabia; ^3^Department of Pathology and Laboratory Medicine, King Abdulaziz Medical City (KAMC), Ministry of National Guard Health Affairs (MNGHA), Riyadh, Saudi Arabia; ^4^World Health Organization, Geneva, Switzerland

**Keywords:** high-risk clones, multidrug resistance, resistome, mobile element, β-lactamase, epidemiology

## Abstract

Carbapenem-resistant *P. aeruginosa* has become a major clinical problem due to limited treatment options. However, studies assessing the trends in the molecular epidemiology and mechanisms of antibiotic resistance in this pathogen are lacking in Saudi Arabia. Here, we reported the genome characterization in a global context of carbapenem non-susceptible clinical isolates from a nationally representative survey. The antibiotic resistance profiles of the isolates (*n* = 635) collected over 14 months between March 2018 and April 2019 from different geographical regions of Saudi Arabia showed resistance rates to relevant β-lactams, aminoglycosides and quinolones ranging between 6.93 and 27.56%. Overall, 22.52% (143/635) of the isolates exhibited resistance to both imipenem and meropenem that were mainly explained by porin loss and efflux overexpression. However, 18.18% of resistant isolates harbored genes encoding GES (69.23%), VIM (23.07%), NDM (3.85%) or OXA-48-like (3.85%) carbapenemases. Most common GES-positive isolates produced GESs −5, −15 or −1 and all belonged to ST235 whereas the VIM-positive isolates produced mainly VIM-2 and belonged to ST233 or ST257. GES and VIM producers were detected at different sampling periods and in different surveyed regions. Interestingly, a genome-wide comparison revealed that the GES-positive ST235 and VIM-2-positive ST233 genomes sequenced in this study and those available through public databases from various locations worldwide, constituted each a phylogenetically closely related sub-lineage. Profiles of virulence determinants, antimicrobial resistance genes and associated mobile elements confirmed relatedness within each of these two different sub-lineages. Sequence analysis located the *bla*_*GES*_ gene in nearly all studied genomes (95.4%) in the same integrative conjugative element that also harbored the *acc(6′)-Ib*, *aph(3′)-XV*, *aadA6*, *sul1*, *tet(G)*, and *catB* resistance genes while *bla*_*VIM*–2_ in most (98.89%) ST233-positive genomes was co-located with *aac(6′)-I1*, *dfrB-5*, and *aac(3′)-Id* in the same class I integron. The study findings revealed the global spread of GES-5 ST235 and VIM-2 ST233 sub-lineages and highlighted the importance of routine detection of rare β-lactamases.

## Introduction

*P. aeruginosa* is a major cause of healthcare associated infections and a serious public health threat due to its ability to resist antibiotics (Gellatly and Hancock, 2013). *P. aeruginosa* is genetically equipped with an outstanding intrinsic antibiotic resistance machinery and is adept at acquiring antibiotic resistance determinants (Lister et al., 2009; López-Causapé et al., 2018). Resistance to carbapenems in the species is due primarily to chromosomal modifications that inactivate or down-regulate the carbapenem-specific OprD porin or modify the expression levels of efflux systems and in particular the MexAB-OprM pump (López-Causapé et al., 2018). In recent years, this pathogen has been increasingly reported as a carrier of acquired carbapenemases and in particular those belonging to the VIM, IMP and GES families (Yoon and Jeong, 2021). *P. aeruginosa* has a non-clonal structure, nonetheless high-risk clones including sequence type (ST)235, ST111, ST233, ST244, ST357, ST308, ST175, ST277, ST654, and ST298 are widespread and frequently associated with outbreaks (Oliver et al., 2015; Miyoshi-Akiyama et al., 2017; del Barrio-Tofiño et al., 2020; Kocsis et al., 2021). ST235 is certainly the most relevant high-risk clone, showing a worldwide dissemination and an association with various β-lactamases, including GES, IMP, KPC, OXA-48, and VIM carbapenemases (Treepong et al., 2018; del Barrio-Tofiño et al., 2020). Other high-risk clones such as ST233, ST357, and ST111 have been also associated with acquired carbapenemases, notably the metallo-β-lactamase VIM, IMP, and NDM types (del Barrio-Tofiño et al., 2020). Using whole genome sequencing, we investigated the mechanisms of resistance to antibiotics in *P. aeruginosa* clinical isolates collected as part of a nationally representative survey from Saudi Arabia and contextualized against a global collection. In addition to porin impairment and overexpression of efflux, sequence analyses showed that resistance to carbapenems was partly due to the clonal spread of GES-5-producing ST235 and VIM-2-producing ST233 sub-lineages. More importantly, a genome-wide comparison revealed that these two sub-lineages were disseminated worldwide.

## Materials and Methods

### Isolates and Phenotypic Characterization

*P. aeruginosa* isolates (*n* = 635) were collected between March-2018 and April-2019 as part of an antimicrobial resistance surveillance program that was initiated in 2018 by the Infectious Diseases Research Department (IDRD) at the National Guard Health Affairs (NGHA) to monitor the prevalence and trends of resistance in a variety of clinically important pathogens. The program involves the monthly collection of the first 10–30 non-duplicate consecutive isolates of each surveyed bacteria identified in the laboratories of NGHA medical cities located in Riyadh, Jeddah, Al Madinah, Dammam and Al Ahsa. The collection included in this study comprised 162 isolates referred from King Abdulaziz Medical City—Riyadh (Centre—Riyadh province, 1,500 bed facility), 275 from King Abdulaziz Medical City—Jeddah (West—Makkah province, 750 bed), 67 from Prince Mohammed Bin Abdul Aziz Hospital—Al Madinah (West—Al Madinah province, 215 bed), 86 from King Abdulaziz Hospital—Al Ahsa (East—Eastern province, 300 bed) and 45 from Imam Abdulrahman Al Faisal Hospital—Dammam (East—Eastern Province, 100 bed). Isolates were recovered from urine (208/635, 32.8%), respiratory (204/635, 32.1%), blood (101/635, 15.9%), wound (67/635, 10.6%) and other specimens (55/635, 8.7%); they were referred at an overall average of 45 (range 19–80) isolates per month (Table 1). Species identification and antimicrobial susceptibility testing were determined with the VITEK II system. Minimum inhibitory concentrations (MICs) of colistin were confirmed using the micro-broth dilution method. MICs were interpreted according to CLSI breakpoints.

**TABLE 1:** Resistance rates to clinically relevant antibiotics among collected isolates.

Regions		Central	Western		Eastern		
City		Riyadh	Jeddah	Al Madinah	Al Ahsa	Dammam	Total
Isolates		162	275	67	86	45	635
Antibiotics	IMI	35.80	28.00	20.90	23.26	13.33	27.56
	MEM	33.95	21.45	20.90	19.77	6.67	23.31
	IMI and MEM	33.33	20.00	20.90	19.77	6.67	22.52
	IMI or MEM	36.42	29.45	20.90	23.26	13.33	28.35
	CAZ	19.14	13.45	11.94	12.79	6.67	14.17
	FEB	10.49	6.90	8.96	11.90	4.44	8.53
	PIP/TAZ	33.75	6.79	19.40	15.29	4.65	16.33
	AMK	9.26	6.18	11.94	2.33	4.44	6.93
	GM	10.49	7.66	13.43	2.35	4.44	8.06
	TOB	10.56	7.14	15.15	0.00	0.00	7.61
	CIP	19.75	16.73	20.90	10.47	4.44	16.22
	CST	2.53	4.52	9.09	1.23	0.00	3.83

*Breakpoints amikacin (AMK) ≥ 64 mg/L; cefepime (FEB) ≥ 32 mg/L; ceftazidime (CAZ) ≥ 32 mg/L; ciprofloxacin (CIP) ≥ 2 mg/L; colistin (COL) ≥ 4 mg/L; piperacillin/tazobactam (PIP/TAZ) ≥ 128/4 mg/L; imipenem (IMI) and meropenem (MER) ≥ 8 mg/L; tobramycin (TOB) and gentamicin (GEN) ≥ 16 mg/L.*

### Species Confirmation and β-Lactamase Screening

Species identity of the isolates was confirmed with a PCR targeting the species-specific *oprL* gene. Presence of genes encoding IMP, GES, KPC, NDM, OXA-48-like, VIM, BEL, PER, and VEB β-lactamases were screened by PCR using the primers described in Supplementary Table 1.

### Whole Genome Sequencing and Bioinformatics

Genomic DNA from all isolates (*n* = 45) was extracted with the MagnaPure compact system (Roche, Switzerland) and prepared for sequencing with the Nextera XT DNA library preparation kit (Illumina, United Kingdom) according to the manufacturer’s instructions. Sequencing was performed on the Miseq instrument using the 2 × 300 paired-end protocol. Of these, nine isolates were further sequenced on the Oxford Nanopore MinION using the ligation sequencing kit according to the manufacturer’s instructions (Oxford Nanopore Technologies, United Kingdom). Genome assemblies using the Illumina reads alone or in combination with the Nanopore long-reads were generated using Unicycler 0.4.8 (Wick et al., 2017). Multilocus sequence type (MLST) was determined *in silico* using the mlst-v2.18.1 software. Genes, mutations associated with antimicrobial resistance, and virulence factors were detected with Abricate 0.9.8^1^ or Genefinder.^2^ In order to put the analysis into an international context, all *P. aeruginosa* paired-end Illumina sequenced genomes (*n* = 16,337) deposited before December 2020 in the NCBI sequence read archive database^3^ were retrieved and the quality of corresponding reads was assessed with the FastQC software. Relatedness of recovered genomes with read coverage above 20x and those generated in this study was inferred using a single nucleotide polymorphisms (SNPs)-based approach by mapping reads against the publically available sequences of strain PAO1 (NC_002516) or the fully closed genome of strain ST235-MPA32 generated in this study. SNPs were first identified with Snippy 4.4.5^4^ to create a full core genome alignment that was latter checked for recombination events using Gubbins 2.4.1 (Croucher et al., 2015). Filtered alignments were then used to construct the phylogenetic trees using RaXML with the default option of Gubbins. Phylogenetic trees were annotated using iTOL v6 and Microreact tools (Argimón et al., 2016; Letunic and Bork, 2021). SNP locations were determined using the annotated genome of the reference strain PAO1 (AE004091.2). Genome assemblies were annotated with prokka 1.14.6 and their gene contents were compared with Roary (Page et al., 2015). The integrative and conjugative elements (ICE) and direct environments of carbapenemases and extended-spectrum β-lactamases in fully closed genomes was determined manually. Presence of these elements in each sequenced genome was later determined by checking the depth of coverage of reads mapped across the full sequence of each mobile element.

## Results

### Isolates and Phenotypic Testing

*P. aeruginosa* clinical isolates were referred over a 14-month period from five hospitals located in the eastern, western and central regions of Saudi Arabia. Susceptibility testing showed that 143/635 (22.52%) isolates were resistant to both imipenem and meropenem (MIC ≥ 8 mg/L) (Table 1). Otherwise, the isolates were variably resistant to ciprofloxacin (16.22%, range 4.44–20.90%), ceftazidime (14.17%, range 6.67–19.14%), cefepime (8.53%, 4.44–11.90%), piperacillin/tazobactam (16.33%, range 4.65–33.75%), amikacin (6.93%, range 2.33–11.94%), gentamicin (8.06%, range 2.35–13.43%) and tobramycin (7.61%, range 0–15.15%) but remained highly sensitive to colistin (96.17%, range 90.91–100%) (Table 1). Resistance to carbapenems was highest in respiratory isolates (40.69%) followed by blood (27.72%), urine (12.02%) then wound (8.96%) swab isolates. Extensively drug-resistant isolates, remaining only sensitive to colistin, accounted for 3.78% (24/635) of all isolates and were predominantly obtained from respiratory specimens (14/24, 58.33%). Regional and individual hospital variations in susceptibility patterns and frequencies were observed for most tested antibiotics (Table 1). Characteristically, hospitals hosting critical patients (i.e., Jeddah and Riyadh) experienced the highest levels of resistance across all tested antibiotics.

### Screening for β-Lactamase Genes in Carbapenem-Resistant Isolates

PCR screening detected genes encoding GES (*n* = 18), VIM (*n* = 6), NDM (*n* = 1), and OXA-48-like (*n* = 1) β-lactamases, explaining resistance to imipenem and meropenem in 18.18% (26/143) of carbapenem-resistant isolates. Genes encoding VEB (*n* = 6) and PER (*n* = 2) extended-spectrum β-lactamases (ESBLs) were detected in only eight isolates, including three of the six VIM-positive isolates. Most common GES- and VIM-positive isolates were referred from all regions at different sampling periods (i.e., 11 out of 14 sampling months) of the survey.

### Genomic Characterization of Carbapenem-Resistant Isolates

To further investigate the molecular mechanisms of resistance to carbapenems, all carbapenemase and ESBL producers identified by PCR (*n* = 31) and a set of randomly selected isolates exhibiting resistance to imipenem and meropenem but with no acquired β-lactamases (*n* = 14) were whole genome sequenced on the Illumina Miseq system.

### GES-Carbapenemase Positive Isolates

**FIGURE 1:**
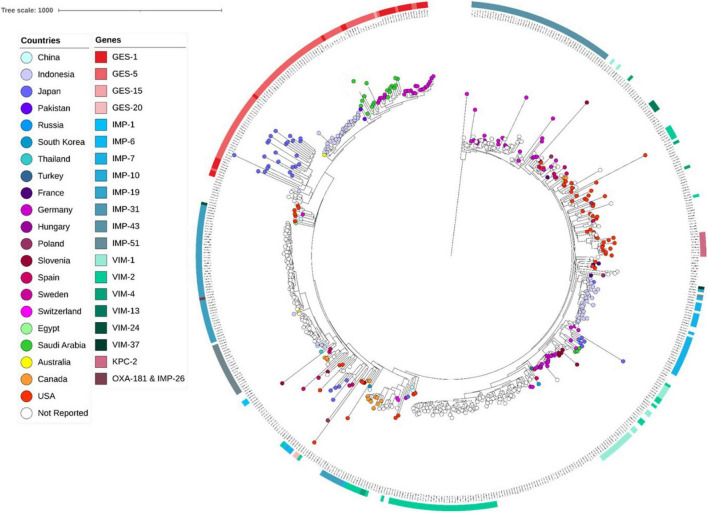
Core genome SNP-based maximum likelihood phylogeny of ST235 genomes retrieved from the public domain and those generated in this study using the genome sequences of strain PAO1 as reference.

**FIGURE 2:**
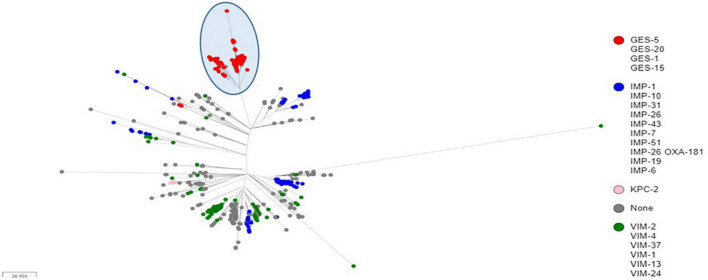
Core genome SNP-based maximum likelihood phylogeny of ST235 genomes retrieved from the public domain and those generated in this study using the MPA32 closed genome as reference. The cluster of GES-positive ST235 genomes were highlighted in a blue circle.

Sequence analyses showed that all genomes carrying genes encoding GES type β-lactamases, including GESs -5 (*n* = 16), −15 (*n* = 1) and −1 (*n* = 1), belonged to ST235. The later accounted for 5.03% (822/16,337) of all genomes retrieved from the public databases, and of which, the majority (71.29%, 586/822) carried genes encoding acquired carbapenemases, including IMP alone (*n* = 264) or in combination with NDM (*n* = 1) or OXA-48-like (*n* = 1), VIM alone (*n* = 133) or in combination with OXA-48-like (*n* = 3), GES (*n* = 174), KPC (*n* = 9), and OXA-48-like (*n* = 1) (Table 2). Full genome SNP-based phylogeny against the *P. aeruginosa* strain PAO1 reference genome grouped nearly all published GES-positive ST235 genomes and those generated in this study in a distinct cluster with the exception of three GES-20 producers which grouped apart (Figure 1). In contrast, the two other common VIM and IMP carbapenemases were distributed across multiple clusters suggesting that they were acquired through multiple events. The phylogenetic tree constructed based on SNP calls relative to the fully closed ST235 MPA32-genome confirmed the clustering of the GES-positive genomes in a well distinct clade (Figure 2). Clustered GES-positive genomes originated from at least six different countries, including Australia, China, Germany, Japan, Indonesia and Pakistan (Figure 1). Phylogeny showed that these producers were related to each other, with at most 120 SNPs to distinguish between them. More specifically, the GES-positive genomes (*n* = 18) from this study clustered tightly with others from Germany (*n* = 26) in a distinct subgroup with at most 45 SNPs to distinguish them from each other. GES-positive genomes were separated from the remaining ST235 genomes by at least 29 SNPs, of which, 14 were non-synonymous in genes classified as transcriptional regulators, metabolic genes and hypothetical proteins whereas four were located in the promoter or potential regulatory regions of genes encoding the porin OprO, global transcriptional regulator IscR or hypothetical proteins (Supplementary Table 2). Of these, only IscR has been shown to regulate genes involved in iron homeostasis, resistance to oxidants and pathogenicity (Romsang et al., 2014; Saninjuk et al., 2019). Comparison of the gene contents of ST235 genomes identified 4,373 core and 1,086 soft-core genes that were shared among 98.94–100% and 94.87–98.94% of genomes studied, respectively. Other 25,228 genes were found to be present within one to a maximum of 536 genomes (i.e., < 94.87%) thus showing a relatively wide genetic variability in the accessory genome of this ST. Overall there were a very limited number of genes (*n* = 27) which presence or absence distinguished the GES-positive cluster from the remaining ST235 but these mainly encoded phage or hypothetical proteins. Only one gene showing homology to the transcriptional repressor NrdR lacked in all GES producers but was present in the majority of remaining ST235 genomes. The latter, shown to regulate the ribonucleotide synthesis may grant the adaptability to thrive in different environments (Crespo et al., 2015). Otherwise, the presence of genomic features encoding the virulence factors gathered in the virulence factor database (VFDB) were comparable across all ST235 genomes. The majority of the genes and gene clusters previously shown to be involved in the species virulence were identified in nearly all (i.e., 98–100%) ST235 genomes with the exception of *wzz* and *wzy* genes that are involved in the B-band lipopolysaccharide O antigen synthesis and the pyoverdine outer membrane receptor *fpvA*. In accordance with previous studies, all ST235 genomes including those carrying *bla*_*GES*_ carried the ExoU toxin-encoding gene (Sato et al., 2003; Treepong et al., 2018).

**TABLE 2:** Distribution of carbapenemase-encoding genes among publically available genomes belonging to the major carbapenemase-positive STs identified in isolates from this study.

ST	β-lactamase type	β-lactamase variant	Nb
ST233 (*n* = 94)	VIM	VIM-2	90
	None		4

ST235 (*n* = 822)	IMP (*n* = 264)	IMP-1	5
		IMP-6	1
		IMP-7	25
		IMP-10	2
		IMP-14	1
		IMP-19	1
		IMP-26	114
		IMP-31	63
		IMP-43	1
		IMP-51	51
	VIM (*n* = 133)	VIM-1	33
		VIM-2	87
		VIM-4	7
		VIM-13	4
		VIM-24	1
		VIM-27	1
	GES (*n* = 174)	GES-1	28
		GES-5	142
		GES-15	1
		GES-20	3
	KPC	KPC-2	9
	OXA-48-like	OXA-232	1
	VIM and OXA	VIM-2 OXA-232	3
	IMP and NDM	IMP-51 NDM-1	1
	IMP and OXA	IMP-26 OXA-181	1
	None		236

ST244 (*n* = 254)	VIM (*n* = 14)	VIM-2	13
		VIM-6	1
	IMP (*n* = 4)	IMP-34	1
		IMP-39	3
	KPC	KPC-2	2
	OXA-48-like	OXA-181	2
	None		232

ST357 (*n* = 229)	IMP (*n* = 46)	IMP-7	42
		IMP-13	2
		IMP-15	1
		IMP-16	1
	NDM	NDM-1	21
	KPC	KPC-2	1
	OXA-48-like	OXA-181	1
	VIM (*n* = 12)	VIM-2	8
		VIM-5	3
		VIM-18	1
	None		148

ST773 (*n* = 23)	VIM	VIM-2	4
	NDM	NDM-1	8
	None		11

**FIGURE 3:**
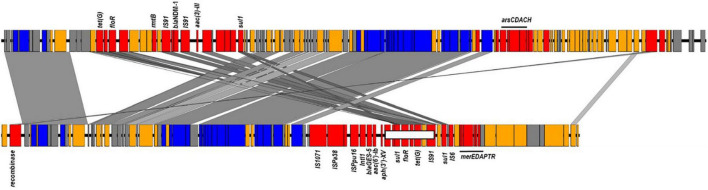
ICEs harboring bla_*NDM*–1_ and bla_*GES*–5_ identified in ST773 and ST235 sequenced genomes. Colors showed in red genes encoding antimicrobial and heavy metal resistance, insertion sequences and transposon-related genes; blue, transfer and conjugative functions; yellow, other known functions and gray, hypothetical proteins. The region carrying the floR and tetG genes missing in some genomes were shown in a white box.

Sequence examination of the fully reconstructed genomes of two GES-5 producers co-located the β-lactamase gene with *acc(6′)-Ib*, *aph(3′)-XV*, *aadA6*, *florR*, *sul1, tetG*, and *catB* genes in a type I integron that was embedded in a 95 kb integrative conjugative elements (ICE) inserted in the chromosome downstream the tRNA-Gly gene (Figure 3). Mapping of the short reads to the full sequence of this element confirmed its presence in nearly all publically available (166/174, 95.4%) and newly sequenced (18/18, 100%) GES-positive genomes. Of the remaining, five (5/174, 2.87%) genomes carrying *bla*_*GES*–1_ (*n* = 1) and *bla*_*GES*–5_ (*n* = 4) lacked the region located between position ∼65.1 and ∼72.1 kb and comprising the *floR* and *tetG* genes whereas the three genome carrying *bla*_*GES*–20_ (3/174, 1.73%) and clustering apart had the entire element missing (Figure 3). One GES-negative isolate (i.e., RPA66) sequenced in this study belonged to ST235 clustered also away, thus confirming the relatedness of the GES-producing ST235 isolates (Supplementary Tables 3, 4).

### VIM-Carbapenemase Positive Isolates

**FIGURE 4:**
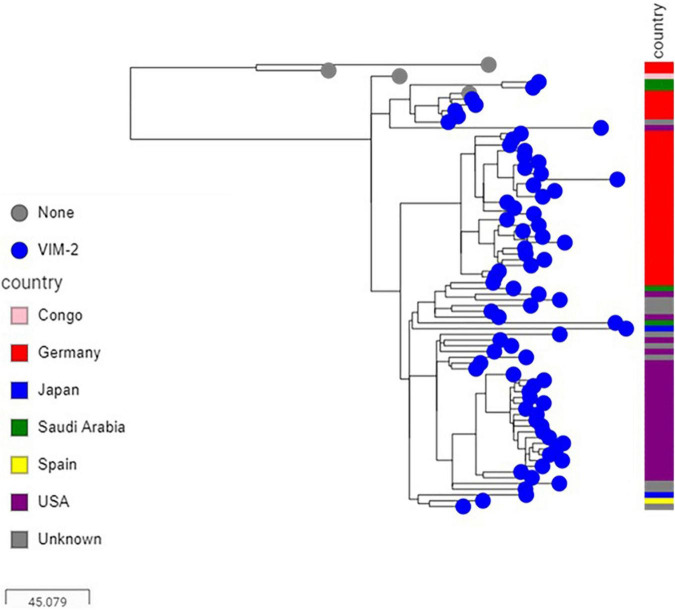
Core genome SNP-based maximum likelihood phylogeny of ST233 genomes retrieved from the public domain and those generated in this study using the genome sequence of strain PAO1 as reference.

**FIGURE 5:**
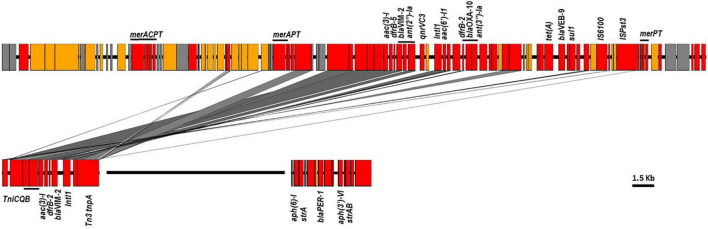
The *bla*_*VIM*–2_, *bla*_*VEB*–9_, and *bla*_*PER*–1_ environments in ST357 (top) and ST233 (bottom) sequenced genomes. Colors showed in red genes encoding antimicrobial and heavy metal resistance, insertion sequences and transposon-related genes; blue, transfer and conjugative functions; yellow, other known functions and gray, hypothetical proteins.

Sequence analysis showed that the majority of VIM-positive genomes (5/6, 83.3%) carried *bla*_*VIM*–2_ and belonged to ST233 (*n* = 3) or ST357 (*n* = 2). The remaining isolate (1/6, 16.7%) harbored *bla*_*VIM*–28_ and belonged to ST111. Isolates carrying *bla*_*VIM*–2_ were from three hospitals located in the three different regions included in the study (Supplementary Table 3). Here also, nearly all published ST233 genomes (90/94, 95.74%), which originated from at least four different countries, including Germany, Japan, Spain and United States, harbored *bla*_*VIM*–2_ and were highly related with at most 47 SNPs to distinguish them from each other. Isolates from this study clustered in two sub groups according to their geographical origins, nevertheless with 29 SNPs to distinguish them from each other (Figure 4). Sequence analysis located the *bla*_*VIM*–2_ of all newly sequenced ST233 isolates in a 4.4 kb class I integron comprising *aac(6′)-I1*, *dfrB-5*, and *aac(3′)-Id* resistance genes (100% identity, accession number AY943084.1), and which, in turn, was embedded in a transposon similar to those found in published *P. aeruginosa* genomes (e.g., CP056774.1 from nucleotide positions 5,258,523–5,270,622) (Figure 5). Screening of publically available *bla*_*VIM*–2_-positive ST233 genomes showed that nearly all (89/90, 98.89%) had reads covering the entire 4.4 kb integron cassette and of which the majority (72/90, 80%) had also reads covering more than 95% of the entire 12 kb transposon. On the other hand, only a handful of publically available ST357 genomes harbored the *bla*_*VIM*–2_ (8/229, 3.49%) and had mainly IMP carbapenemases (46/229, 20.09%) and in particular the IMP-7 variant (42/46, 95.45%). The *bla*_*VIM*–2_ of newly sequenced ST357 genomes was detected in an integron type I mobile element embedded in a complex transposon containing all the resistance genes identified in this isolate (Figure 5). In contrast, the *bla*_*VIM*–28_ in the ST111 isolate was located on a 350 kb plasmid in a integron type I in association with *aac(6′)-I*.

### Other Carbapenemase-Positive Isolates

Of the remaining carbapenemase-positive isolates, one carried *bla*_*OXA*–232_ and belonged to ST244 while one had *bla*_*NDM*–1_ and belonged to ST773. Genome assemblies located the *bla*_*OXA*–232_ gene on a 6.1 kb ColKP3-type non-conjugative plasmid similar to previously published plasmid pColKP3 (accession number CP036323). Otherwise, the *bla*_*NDM*–1_ was co-located with *floR2*, *rmtB*, *sul1*, and *tet(G)* in an ICE element of approximately ∼117 kb that was highly similar to the clc-like ICE recently identified in *P. aeruginosa* (accession number MK497171) (Figure 3).

### Molecular Mechanisms of Resistance

#### β-Lactams

In addition to the acquisition of carbapenemases, sequence analysis identified alterations in the outer membrane protein OprD leading to function loss in the majority (82.22%, 37/45) of sequenced genomes. Porin alterations included large deletions at the beginning or end of the coding region (*n* = 11) and frameshifts produced by small insertions or deletions creating premature translational termination at various positions (*n* = 26) (Supplementary Table 4). Genes encoding the MexAB-OprM efflux system were highly conserved across all sequenced isolates. However, inactivation of either MexR (*n* = 4), NalC (*n* = 1), or NalD (*n* = 9), previously shown to up-regulate the expression of this efflux pump, was identified in nearly third (14/45, 31.1%) of sequenced isolates and mainly in the non-carbapenemase producers (9/14, 64.3%) (Supplementary Tables 3, 4). Inactivation of these regulators was due to various insertions, deletions, or substitutions resulting in frameshift of the reading frame, and creating premature stop codons (Supplementary Tables 3, 4). Overall, the acquisition of carbapenemase genes with porin inactivation and efflux upregulation explained resistance to imipenem and meropenem in all sequenced genomes. Carbapenemase-producing isolates (*n* = 26) were resistant to all tested non-carbapenem β-lactams (i.e., piperacillin/tazobactam, cefipime, and ceftazidime) with the exception of those producing GES-5, which remained in majority (10/18, 55.56%) sensitive or intermediately resistant to ceftazidime (MIC 16 mg/L) and cefepime (MIC 8–16 mg/L). Resistance in some of these producers suggested an overexpression of the chromosomal AmpC but none had mutations in the coding sequences of AmpR, AmpRh1, AmpRh2, AmpD, PBPs, and GalU or in the *ampR*-*ampC* intergenic region to explain resistance. Of the remaining non-carbapenemase producers (*n* = 20), only six were resistant to ceftazidime (MIC ≥ 64 mg/L) and harbored GES-1 (*n* = 1), VEB (*n* = 4), or PER (*n* = 1) ESBL variants (Supplementary Tables 3, 4).

#### Other Antibiotics

Scanning genome sequences identified alterations in GyrA (T83I/T83A and D87Y) and ParC (S87L) in all isolates (33/45, 73.33%) showing high level of resistance to ciprofloxacin (MIC ≥ 4 mg/L). Only three of the seven isolates exhibiting low levels of resistance (MIC 1–2 mg/L) carried the plasmid-encoded ciprofloxacin resistance determinant *crpP* or had the MexAB-OprM efflux pump overexpressed (Supplementary Tables 3, 4). However, the presence of *crpP* was detected in nearly half of the isolates (22/45, 48.89%) including in two sensitive isolates (MIC ≤ 0.25 mg/L). Resistance to all three tested aminoglycosides was associated in the majority of cases (*n* = 18) with the presence of the aminoglycoside-modifying enzyme Aac(6′)-Ib-cr and overexpression of the MexXY-OprM efflux system due to inactivation of its repressor MexZ by a deletion of 11 nucleotides creating a translational frameshift at position 290. Presence of the acquired *aac(6′)-I*, *aac(3′)-I*, *ant(2″)-Ia*, or the 16S rRNA methyltransferase *rmtB* genes explained resistance in 11 isolates while one isolate carried *acc(3′)-I* and had the MexXY-OprM overexpressed due to inactivation of MexZ by a substitution creating a premature stop codon at position 162 (Supplementary Tables 3, 4). Resistance in three isolates did not correlate with the genomic data. Otherwise, the borderline resistance to colistin (MIC 4 mg/L) in few isolates (*n* = 6) were not associated with any mutations in the PmrA/PmrB or PhoP/PhoQ two-component systems (Supplementary Tables 3, 4).

## Discussion

Susceptibility testing of *P. aeruginosa* clinical isolates from a large, nationally representative collection of Saudi Arabia showed an overall resistance rates to relevant aminoglycosides, quinolones, and β-lactams ranging from 6.93 to 27.56%. Resistance to either imipenem or meropenem varied greatly among recruited hospitals (28.35%, range 13.33–36.42%) with the highest levels of resistance observed in central region (i.e., Riyadh tertiary hospital). Sequence analysis identified loss of porin OprD and efflux overexpression at the origin of resistance in the majority (i.e., 81.56%) of sequenced isolates. Of the four efflux systems of the resistance nodulation division (RND) family, MexAB-OprM, MexEF-OprN, MexCD-OprJ, and MexXY-OprM that are known to contribute to antimicrobial resistance in the species, sequence analyses suggested that overexpression of the MexAB-OprM efflux, mainly through inactivation of its regulatory genes, constitute the main system acting synergistically with low outer membrane permeability to confer intrinsic multi-drug resistance (Poole et al., 1993, 1996; Köhler et al., 1997; Mine et al., 1999). More importantly, resistance to carbapenems was partly (i.e., 18.18%) associated with the acquisition of acquired carbapenemases, notably those encoding the GES and VIM type enzymes. GES-5 producers, which all belonged to ST235, were by far the most dominant among resistant isolates carrying acquired carbapenemases; they were also widespread, being identified in all regions surveyed during the study period. A previous study suggested that the ST235 lineage emerged in Europe but have since evolved globally and acquired locally diverse antimicrobial resistance determinants (Abril et al., 2019). The genome-wide sequence analysis identified a high genomic diversity among ST235 isolates and confirmed local acquisitions of carbapenemase-encoding genes. However, the GES-positive ST235 genomes sequenced in this study and all those publically available were similar and belonged to the same phylogenetic group. Moreover, the GES-encoding genes in nearly all these genomes (95.4%) was located in an identical ICE, thus supporting the early acquisition of the β-lactamase gene in this sub-lineage. The association of this ST with various β-lactamases has been in part linked to the presence of type IV secretion systems promoting foreign DNA capture, leading to the insertion of genetic element as transposon, integron, or genomic islands harboring resistance genes (Miyoshi-Akiyama et al., 2017; Treepong et al., 2018). Gene by gene comparisons identified only one gene showing homology to the regulator of deoxyribonucleotide reduction NdrD that was missing in the GES-positive genomes but present in the majority of remaining genomes. However, the significance of the absence of this gene need to be further investigated. Similar findings were also observed for the VIM-2-positive ST233 isolates where genome comparisons revealed that sequenced genomes and those publically available were phylogenetically similar to each other. In contrast to ST235, nearly all ST233 genomes (90/94, 95.75%) in the public domain carried the VIM-2-encoding gene. Here also the environment of the β-lactamase gene, which was co-located in the same class I integron in all genomes, supported an ancestral acquisition and subsequent spreading, rather than multiple acquisition events.

The molecular basis of extended-spectrum β-lactamase (ESBL) and carbapenemase production in *P. aeruginosa* isolated from Saudi Arabia was reported in a limited number of studies but none has been based on whole-genome sequencing. Overall, these reports indicated that VIM-type enzymes were the most prevalent metallo-β-lactamase in isolates from the Kingdom (Al-Agamy et al., 2009; Shaaban et al., 2018). Detection of genes encoding the VIM-2 variant in ST233 isolates has been reported in several countries worldwide, including in a handful of isolates from Saudi Arabia and neighboring Bahrain and Egypt (Zafer et al., 2015; Zowawi et al., 2018). A recent study reported the identification of VIM-2 in isolates belonging to ST654 and GES in ST235 isolates from one hospital located in the western region (Al-Zahrani and Al-Ahmadi, 2021). Although VEB-like enzymes were the most common reported ESBLs in *P. aeruginosa* isolates from Saudi Arabia, few studies reported the detection of GES encoding genes in isolates from the Kingdom (Al-Agamy et al., 2012, 2016; Tawfik et al., 2012).

Overall, the study findings clearly showed a worldwide dissemination of the GES-5-producing ST235 and VIM-2-producing ST233 sub-lineages. Moreover, a recent study reporting the spread of ST235 isolates producing GES-type enzymes across multiple regions in Japan, confirmed the potential of these lineages to disseminate broadly (Hishinuma et al., 2018). The study results also emphasize the fact that the spread of strains producing rare carbapenemases, such as GES-type enzymes, could be underestimated because the genes encoding these β-lactamases are outside the scope of all commercially available assays that are mainly focused on the detection of most common NDM, VIM, OXA-48-like, KPC and IMP carbapenemase genes, and thus highlighting the importance of screening for these β-lactamases.

## Data Availability Statement

The datasets presented in this study can be found in online repositories. The names of the repository/repositories and accession number(s) can be found below: https://www.ncbi.nlm.nih.gov/bioproject/PRJNA751257.

## Author Contributions

MD, HB, SAJ, LO, and MFA contributed to the design of the study. The NGHA antimicrobial surveillance group collected the samples on a monthly basis. EA led the initial laboratory processing of the samples. SAH performed the β-lactamase screening by PCR. MAZ, SAH, and LO performed the genome sequencing. MD and AA performed the genomic data analysis. MD led the writing and revision of the manuscript. All authors commented and contributed on the final version of the manuscript.

## Conflict of Interest

HB has participated in this work during her tenure as Professor of pediatric infectious diseases at King Saud bin Abdulaziz University for Health Sciences. The remaining authors declare that the research was conducted in the absence of any commercial or financial relationships that could be construed as a potential conflict of interest.

## Publisher’s Note

All claims expressed in this article are solely those of the authors and do not necessarily represent those of their affiliated organizations, or those of the publisher, the editors and the reviewers. Any product that may be evaluated in this article, or claim that may be made by its manufacturer, is not guaranteed or endorsed by the publisher.

## Footnotes

1
https://github.com/tseemann/abricate

2
https://github.com/phe-bioinformatics/gene_finder

3
https://www.ncbi.nlm.nih.gov/sra

4
https://github.com/tseemann/snippy

## NGHA AMR Surveillance Group

Majed F. Alghoribi, Liliane Okdah, Essa Alrashidi, Alhanouf Alshahrani, Sameera Aljohani, Bassam Alalwan, Abdulfattah Al-Amri, Mai M. Kaaki, Mohamed Doud, Haitham S. Dadah, Fahad Alnashmy, and Michel Doumith.
