# Supplementary material for: Genomic Characterization of Carbapenem-Non-susceptible Pseudomonas aeruginosa Clinical Isolates From Saudi Arabia Revealed a Global Dissemination of GES-5-Producing ST235 and VIM-2-Producing ST233 Sub-Lineages
Source: Front Microbiol. 2022 Jan 6;12:765113. doi: 10.3389/fmicb.2021.765113 (PMC8770977; doi:10.3389/fmicb.2021.765113)
Supplement: Supplementary file 1 [file Table_1.docx]

Supplementary Table 1. Primers used for species identification and detection of β-lactamase genes.

| Gene | Primer id | Primer sequence | Amplicon size (bp) |
| --- | --- | --- | --- |
| *bla*_VEB_ | VEB-F | CCCGATGCAAAGCGTTATGA | 642 |
|  | VEB-R | CGAAGTTTCTTTGGACTCTG |  |
| *bla*_GES_ | GES-F | AGTCGGCTAGACCGGAAAG | 399 |
|  | GES-R | TTTGTCCGTGCTCAGGAT |  |
| *bla*_PER_ | PER-F | GCTCCGATAATGAAAGCGT | 520 |
|  | PER-F1 | TCGCCTATGATGAAAGATC |  |
|  | PER-R1 | TTCGGCTTGACTCGGCTGA |  |
|  | PER-R | TTCGGCTTGACTCGGCTGA |  |
| *bla*_BEL_ | BEL-F | GCTCTACCCGTTATTGCTGT | 783 |
|  | BEL-R | CGCCTTGCAATTCAGGTGC |  |
| *bla*_NDM_ | NDM-F | ACCGAATGTCTGGCAGCACA | 625 |
|  | NDM-R | GGGCCGTATGAGTGATTGC |  |
| *bla*_KPC_ | KPC-F | TCTGCTGTCTTGTCTCTCATG | 790 |
|  | KPC-R | CTTGTCATCCTTGTTAGGCG |  |
| *bla*_OXA-48-like_ | OXA-F | GGTTAAGGATGAACACCAAGTC | 508 |
|  | OXA-R | TTGTGATGGCTTGGCGCAG |  |
| *bla*_IMP_ | IMP-F1 | GGAATAGGGTGGCTTAATTCTC | 190 |
|  | IMP-F2 | GGAATAGAGTGGCTTAACTCTC |  |
|  | IMP-F3 | GGAATTGAGTGGCTTAATTCTC |  |
|  | IMP-F4 | GGAATAGAGTGGCTTAATTCTC |  |
|  | IMP-F5 | GGAATAGAATGGCTTAACTCTC |  |
|  | IMP-R1 | CCAAACCACTACGTTATCTTG |  |
|  | IMP-R2 | CCAAACCACTACGTTATCTGG |  |
|  | IMP-R3 | CCAAACCACTAGGTTATCTTG |  |
|  | IMP-R4 | CCAAACTACTAGGTTATCTGG |  |
|  | IMP-R5 | CCAAACTACTACGTTGTCTTG |  |
|  | IMP-R6 | CCAAACCACTACATTATCCTG |  |
| *oprL* | OPR-F | CGATGCTTCCGGTGAAGGT | 230 |
|  | OPR-R | GTGTGGCCTTCCAGCACTA |  |
