# Supplementary material for: Genomic Characterization of Carbapenem-Non-susceptible Pseudomonas aeruginosa Clinical Isolates From Saudi Arabia Revealed a Global Dissemination of GES-5-Producing ST235 and VIM-2-Producing ST233 Sub-Lineages
Source: Front Microbiol. 2022 Jan 6;12:765113. doi: 10.3389/fmicb.2021.765113 (PMC8770977; doi:10.3389/fmicb.2021.765113)
Supplement: Supplementary file 2 [file Table_2.docx]

Supplementary Table 2. Mutations differentiating the GES-positive ST235 from other ST235 genomes.

| SNP | Position | Element | Location | Gene function |
| --- | --- | --- | --- | --- |
| C > T | 11620 | Gene* | Complement (10434..12488) | glyS: glycyl-tRNA synthetase beta chain |
| C > T | 11621 | Gene* | Complement (10434..12488) | glyS: glycyl-tRNA synthetase beta chain |
| T > A | 65898 | Regulatory* | 66303..68135 | phzH: potential phenazine-modifying enzyme |
| G > T | 389823 | Gene# | Complement (389733..390884) | glpQ: glycerophosphoryl diester phosphodiesterase |
| C > T | 427988 | Gene# | Complement (427586..428740) | probable oxidase |
| C > T | 548141 | Gene# | Complement (547432..548406) | hypothetical protein |
| T > G | 841311 | Gene* | 841079..841825 | pdxJ: pyridoxal phosphate biosynthetic protein |
| G > T | 941182 | Gene* | 940117..942573 | hypothetical protein |
| T > C | 1119918 | Gene* | Complement (1119674..1122217) | quiP: acilase |
| C > G | 1152699 | Gene* | 1152624..1153538 | probable transcriptional regulator |
| G > C | 1219078 | Gene# | Complement (1218940..1219527) | hypothetical protein |
| A > G | 1383522 | Gene* | 1381804..1383654 | probable TonB-dependent receptor |
| G > A | 2703048 | Gene* | 2702926..2704344 | probable porin |
| G > A | 2950079 | Gene# | 2949967..2950950 | hypothetical protein |
| C > G | 3356485 | Gene* | Complement (3356162..3357499) | nqrA: Na(+)-translocating NADH-quinone reductase |
| G > A | 3554229 | Gene* | Complement (3554157..3555254) | pheA: chorismate mutase |
| C > A | 3674553 | Regulatory* | Complement (3673008..3674324) | oprO: outer membrane porin OprO precursor |
| C > A | 3870270 | Gene# | 3869461..3870657 | hypothetical protein |
| C > T | 4229142 | Gene# | 4229050..4230027 | probable transcriptional regulator |
| C > G | 4260560 | Gene* | Complement (4260399..4261043) | hypothetical protein |
| C > A | 4272702 | Regulatory* | Complement (4272165..4272656) | iscR |
| C > A | 4530489 | Gene# | Complement (4529995..4530612) | ribA: GTP cyclohydrolase II protein |
| G > A | 4644978 | Gene* | 4644578..4645597 | acoB: acetoin catabolism protein AcoB |
| G > A | 4681934 | Gene* | 4681421..4682149 | probable transcriptional regulator |
| A > G | 4770073 | Gene# | Complement (4769035..4771155) | fusA1: elongation factor G |
| C > T | 4866337 | Regulatory* | Complement (4865682..4866293) | hypothetical protein |
| G > A | 5178116 | Gene* | Complement (5177655..5178902) | probable cytochrome C-type |
| G > A | 5244605 | Gene# | 5243178..5245406 | probable TonB-dependent receptor |
| G > T | 6120857 | Gene* | 6119961..6120896 | probable transcriptional regulator |

Gene location and annotation are based on the GenBank of strain PAO1 (accession AE004091.2).

(*) indicated mutations detected only in the GES-producing ST235 genomes (#) indicated mutations detected in non-GES-producing ST235 genomes.
