# Supplementary material for: Genomic Characterization of Carbapenem-Non-susceptible Pseudomonas aeruginosa Clinical Isolates From Saudi Arabia Revealed a Global Dissemination of GES-5-Producing ST235 and VIM-2-Producing ST233 Sub-Lineages
Source: Front Microbiol. 2022 Jan 6;12:765113. doi: 10.3389/fmicb.2021.765113 (PMC8770977; doi:10.3389/fmicb.2021.765113)
Supplement: Supplementary file 3 [file Table_3.docx]

Supplementary Table 3. Characteristics of sequenced isolates from this study.

| Isolate | City | Date | Specimen | Antibiotic MICs (mg/L) | | | | | | | | | |
| --- | --- | --- | --- | --- | --- | --- | --- | --- | --- | --- | --- | --- | --- |
| Isolate | City | Date | Specimen | PIP/TAZ | CAZ | FEP | IMP | MEM | AMK | GM | TOB | CIP | COL |
| RPA78 | Riyadh | Oct-18 | Urine | ≥128 | ≥64 | ≥64 | ≥16 | ≥16 | ≥64 | ≥16 | ≥16 | ≥4 | 1 |
| JPAB28 | Jeddah | Oct-18 | Blood | ND | ≥64 | ≥64 | ≥16 | ≥16 | ≥64 | ≥16 | ≥16 | ≥4 | ≤0.5 |
| JPAR60 | Jeddah | Oct-18 | Respiratory | ND | ≥64 | ≥64 | ≥16 | ≥16 | ≥64 | ≥16 | ≥16 | ≥4 | 1 |
| RPA10 | Riyadh | Mar-18 | Respiratory | ≥128 | ≥64 | ≥64 | ≥16 | ≥16 | ≥64 | ≥16 | ≥16 | ≥4 | 4 |
| RPA85 | Riyadh | Nov-18 | Respiratory | ≥128 | ≥64 | ≥64 | ≥16 | ≥16 | ≥64 | ≥16 | ≥16 | ≥4 | 2 |
| JPAR31 | Jeddah | Jul-18 | Respiratory | ≥128 | ≥64 | 32 | ≥16 | ≥16 | ≥64 | ≥16 | ≥16 | ≥4 | ≤0.5 |
| JPAR65 | Jeddah | Oct-18 | Respiratory | ≥128 | ≥64 | 32 | ≥16 | ≥16 | ≥64 | ≥16 | ≥16 | ≥4 | ≤0.5 |
| JPAR79 | Jeddah | Dec-18 | Respiratory | ND | 16 | 8 | ≥16 | ≥16 | ≥64 | ≥16 | ≥16 | ≥4 | ≤0.5 |
| JPAU63 | Jeddah | Dec-18 | Urine | ≥128 | 16 | 8 | ≥16 | ≥16 | ≥64 | ≥16 | ≥16 | ≥4 | ≤0.5 |
| MPA01 | Al Madinah | Mar-18 | Urine | 32 | 16 | 8 | ≥16 | ≥16 | ≥64 | ≥16 | ≥16 | ≥4 | ≤0.5 |
| MPA31 | Al Madinah | Jul-18 | Respiratory | ≥128 | ≥64 | 32 | ≥16 | ≥16 | ≥64 | ≥16 | ≥16 | ≥4 | ≤0.5 |
| MPA32 | Al Madinah | Aug-18 | Respiratory | ≥128 | 16 | 8 | ≥16 | ≥16 | ≥64 | ≥16 | ≥16 | ≥4 | ≤0.5 |
| MPA54 | Al Madinah | Nov-18 | Wound | 32 | 16 | 8 | ≥16 | ≥16 | ≥64 | ≥16 | ≥16 | ≥4 | ≤0.5 |
| RPA109 | Riyadh | Nov-18 | Blood | ≥128 | 16 | 16 | ≥16 | ≥16 | ≥64 | ≥16 | ≥16 | ≥4 | 2 |
| RPA128 | Riyadh | Jan-19 | Blood | ≥128 | 16 | 8 | ≥16 | ≥16 | ≥64 | ≥16 | ≥16 | ≥4 | ≤0.5 |
| RPA185 | Riyadh | Mar-19 | Respiratory | ≥128 | 16 | 8 | ≥16 | ≥16 | ≥64 | ≥16 | ≥16 | ≥4 | ≤0.5 |
| RPA206 | Riyadh | Apr-19 | Respiratory | ≥128 | ≥64 | 16 | ≥16 | ≥16 | ≥64 | ≥16 | ≥16 | ≥4 | ≤0.5 |
| RPA23 | Riyadh | May-18 | Blood | ≥128 | 16 | 8 | ≥16 | ≥16 | ≥64 | ≥16 | ≥16 | ≥4 | ≤0.5 |
| RPA32 | Riyadh | Jun-18 | Urine | ≥128 | ≥64 | 16 | ≥16 | ≥16 | ≥64 | ≥16 | ≥16 | ≥4 | 2 |
| RPA41 | Riyadh | Jul-18 | Respiratory | ≥128 | 32 | 16 | ≥16 | ≥16 | ≥64 | ≥16 | ≥16 | ≥4 | 2 |
| RPA66 | Riyadh | Oct-18 | Urine | ≥128 | ≥64 | ≥64 | 8 | ≥16 | 16 | ≥16 | ≥16 | ≥4 | ≤0.5 |
| RPA91 | Riyadh | Nov-18 | Respiratory | ≥128 | ≥64 | 32 | ≥16 | 8 | ≥64 | ≥16 | ≥16 | ≥4 | ≤0.5 |
| JPAB50 | Jeddah | Jan-19 | Blood | ≥128 | 1 | 32 | ≥16 | ≥16 | 4 | ≤ 1 | ≤ 1 | ≤ 0.25 | 4 |
| JPAU54 | Jeddah | Nov-18 | Urine | 16 | 4 | 4 | ≥16 | ≥16 | ≥64 | ≥16 | ≥16 | ≥4 | 2 |
| DPA57 | Dammam | Jan-19 | Respiratory | ≥128 | ≥64 | ≥64 | ≥16 | ≥16 | ≥64 | ≥16 | ≥16 | ≥4 | ≤0.5 |
| JPAB41 | Jeddah | Dec-18 | Blood | ND | ≥64 | ≥64 | ≥16 | ≥16 | 16 | 8 | ≥16 | ≥4 | ≤0.5 |
| RPA135 | Riyadh | Jan-19 | Respiratory | ≥128 | ≥64 | ≥64 | ≥16 | ≥16 | ≥64 | ≥16 | ≥16 | ≥4 | ≤0.5 |
| RPA226 | Riyadh | Apr-19 | Blood | ≥128 | ≥64 | ≥64 | ≥16 | ≥16 | ≥64 | ≥16 | ≥16 | ≥4 | ≤0.5 |
| RPA37 | Riyadh | Jul-18 | Blood | ≥128 | 8 | 4 | ≥16 | 8 | ≤2 | ≤1 | ≤ 1 | ≤ 0.25 | ≤0.5 |
| DPA32 | Dammam | Oct-18 | Blood | ND | 4 | 2 | ≥16 | 8 | 4 | ≤1 |  | 0.5 | 2 |
| MPA91 | Al Madinah | Apr-19 | Respiratory | ≥128 | ≥64 | ≥64 | ≥16 | ≥16 | ≥64 | ≥16 | ≥16 | ≥4 | ≤0.5 |
| JPAB38 | Jeddah | Dec-18 | Blood | 32 | 16 | 4 | ≥16 | ≥16 | 16 | 8 | 4 | ≥4 | 4 |
| JPAR102 | Jeddah | Feb-19 | Respiratory | 16 | 4 | 4 | ≥16 | ≥16 | ≤ 2 | ≤ 1 | ≤ 1 | 1 | 4 |
| JPAO31 | Jeddah | Nov-18 | Others | ND | 4 | ≤ 1 | 1 | ≥16 | 4 | ≤ 1 | ≤ 1 | 0.5 | ≤0.5 |
| RPA100 | Riyadh | Nov-18 | Respiratory | ≥128 | 16 | 8 | ≥16 | ≥16 | 8 | 2 | ≤1 | 1 | 4 |
| RPA117 | Riyadh | Dec-18 | Blood | ND | 4 | 4 | ≥16 | ≥16 | 4 | ≤1 | 4 | 2 | 1 |
| JPAB24 | Jeddah | Sep-18 | Blood | 8 | 4 | 4 | 1 | ≤0.25 | ≤ 2 | 2 | ≤ 1 | ≥4 | ≤0.5 |
| JPAB21 | Jeddah | Sep-18 | Blood | 32 | 16 | 16 | ≥16 | ≥16 | 16 | 8 | ≤ 1 | 1 | ≤ 0.5 |
| JPAU51 | Jeddah | Nov-18 | Urine | ND | 16 | 8 | ≥16 | ≥16 | ≤ 2 | ≥16 | ≥16 | ≤0.25 | ≤0.5 |
| MPA14 | Al Madinah | May-18 | respiratory | ≥128 | 16 | 16 | ≥16 | ≥16 | ≥64 | ≥16 | ≥16 | ≥4 | ≤0.5 |
| JPAU94 | Jeddah | Apr-19 | Urine | ≥128 | ≥64 | 16 | ≥16 | ≥16 | ≥64 | ≥16 | ≥16 | ≥4 | ≤0.5 |
| HPA69 | Al Ahsa | Jan-19 | Urine | 8 | 4 | 8 | ≥16 | 8 | ≥64 | 8 | 4 | 2 | 4 |
| RPA61 | Riyadh | Sep-18 | Respiratory | ≥128 | 16 | 8 | ≥16 | ≥16 | 4 | 4 | 2 | 2 | 2 |
| JPAU32 | Jeddah | Aug-18 | Urine | ≥128 | ≥64 | ≥64 | ≥16 | ≥16 | ≥64 | ≥16 |  | ≥4 |  |
| DPA39 | Dammam | Oct-18 | Blood | 8 | 4 | 2 | 1 | ≤0.25 | ≤ 2 | 2 | ≤ 1 | 1 | ≤ 0.5 |

AMK, amikacin; CAZ, ceftazidime; CIP, ciprofloxacin; COL, colistin; FEB, cefepime; GEN, gentamicin; IMI, imipenem; MER, meropenem; PIP/TAZ, piperacillin/tazobactam; TOB, tobramycin; ND, not determined.
