# Supplementary material for: Genomic Characterization of Carbapenem-Non-susceptible Pseudomonas aeruginosa Clinical Isolates From Saudi Arabia Revealed a Global Dissemination of GES-5-Producing ST235 and VIM-2-Producing ST233 Sub-Lineages
Source: Front Microbiol. 2022 Jan 6;12:765113. doi: 10.3389/fmicb.2021.765113 (PMC8770977; doi:10.3389/fmicb.2021.765113)
Supplement: Supplementary file 4 [file Table_4.docx]

Supplementary Table 4. Sequence types, chromosomal mutations and acquired genes associated with resistance to antibiotics in sequenced isolates.

| Isolate  (ST) | β-lactamase | Impermeability | | MexAB-OprM | | MexXY-OprM | | GyrA | | ParC | | Acquired resistance determinants | |
| --- | --- | --- | --- | --- | --- | --- | --- | --- | --- | --- | --- | --- | --- |
|  |  | nuc | pro | nuc | pro | nuc | pro | nuc | pro | nuc | pro | aminoglycosides | others |
| RPA78#  (ST111) | *bla*_VIM-28_ | *oprD*: Ins 742 (CCGAC) | 346* | *mexR*: Ins 218 (CC) | F | *mexZ*: T486G | 162* | C248T | T83I | C260T | S87L | *aac(3)-Ie* | *catB, sul1, crpP* |
| JPAB28  (ST233) | *bla*_OXA-33_ *bla*_PER-1_ | *oprD*: del 631 (G) | 229* | *mexR*: del 97-260 | T | - | - | C248T | T83I | C260T | S87L | *aac(3)-Id, aac(6')-I, aadA-2, aadA17, aph(3')-VIb, strAB* | *dfrB5, tetG, catB, cml, sul1, crpP* |
| JPAR60#  (ST233) | *bla*_VIM-2_ *bla*_OXA-33_ *bla*_PER-1_ | *oprD*: del 631 (G) | 229* | *mexR*: del 97-261 | T | - | - | C248T | T83I | C260T | S87L | *aac(3)-Id, aac(6')-I, aadA-2, aadA8b, aph(3')-VIb, strAB* | *dfrB5, tetG, catB, cml, sul1, crpP* |
| RPA10#  (ST233) | *bla*_VIM-2_ *bla*_OXA-33_ | *oprD*: del 846 (CTACACTCTGGAT) | 341* | - | - | - | - | C248T | T83I | C260T | S87L | *aac(3)-Id, aac(6')-I, aadA-2, aadA17* | *dfrB5, tetG, catB, cml, sul1, crpP* |
| RPA85#  (ST233) | *bla*_VIM-2_ *bla*_OXA-4_ | *oprD*: del 329-1332 | - | - | - | - | - | C248T | T83I | C260T | S87L | *aac(3)-Id, aac(6’)-I, aadA-2, aadA17* | *dfrB5, tetG, catB, sul1, crpP* |
| JPAR31  (ST235) | *bla*_GES-1_ | - | - | - | - | *mexZ*: del 293 (CGAGCCCGGTT) | F | C248T | T83I | C260T | S87L | *aac(6')-Ib-cr, aadA-6, aph(3')-XV* | *tetG, catB, florR, sul1* |
| JPAR65  (ST235) | *bla*_GES-5_ | *oprD*: del 89 (AGGCCGAAGCGAA) | 90* | - | - | *mexZ*: del 293 (CGAGCCCGGTT) | F | C248T | T83I | C260T | S87L | *aac(6')-Ib-cr, aadA-6, aph(3')-XV* | *tetG, catB, florR, sul1* |
| JPAR79  (ST235) | *bla*_GES-5_ | *oprD*: G195A | 64* | - | - | *mexZ*: del 293 (CGAGCCCGGTT) | F | C248T | T83I | C260T | S87L | *aac(6')-Ib-cr, aadA-6, aph(3')-XV* | *tetG, catB, florR, sul1* |
| JPAU63  (ST235) | *bla*_GES-5_ | *oprD*: G17A | 6* | - | - | *mexZ*: del 293 (CGAGCCCGGTT) | F | C248T | T83I | C260T | S87L | *aac(6')-Ib-cr, aadA-6, aph(3')-XV* | *tetG, catB, florR, sul1* |
| MPA01  (ST235) | *bla*_GES-5_ | *oprD*: del 89 (AGGCCGAAGCGAA) | 90* | - | - | *mexZ*: del 293 (CGAGCCCGGTT) | F | C248T | T83I | C260T | S87L | *aac(6')-Ib-cr, aadA-6, aph(3')-XV* | *tetG, catB, florR, sul1, crpP* |
| MPA31  (ST235) | *bla*_GES-5_ | - | - | *nalD*: del 489-639 | T | *mexZ*: del 293 (CGAGCCCGGTT) | F | C248T | T83I | C260T | S87L | *aac(6')-Ib-cr, aadA-6, aph(3')-XV* | *tetG, catB, florR, sul1, crpP* |
| MPA32#  (ST235) | *bla*_GES-5_ | - | - | - | - | *mexZ*: del 293 (CGAGCCCGGTT) | F | C248T | T83I | C260T | S87L | *aac(6')-Ib-cr, aadA-6, aph(3')-XV* | *tetG, catB, sul1, crpP* |
| MPA54#  (ST235) | *bla*_GES-5_ | *oprD*: del 89 (AGGCCGAAGCGAA) | 90* | - | - | *mexZ*: del 293 (CGAGCCCGGTT) | F | C248T | T83I | C260T | S87L | *aac(6')-Ib-cr, aadA-6, aph(3')-XV* | *tetG, catB, florR, sul1, crpP* |
| RPA109  (ST235) | *bla*_GES-5_ | *oprD*: del 1-899 | - | - | - | *mexZ*: del 293 (CGAGCCCGGTT) | F | C248T | T83I | C260T | S87L | *aac(6')-Ib-cr, aadA-6, aph(3')-XV* | *tetG, catB, florR, sul1* |
| RPA128  (ST235) | *bla*_GES-5_ | *oprD*: del 1-899 | - | - | - | *mexZ*: del 293 (CGAGCCCGGTT) | F | C248T | T83I | C260T | S87L | *aac(6')-Ib-cr, aadA-6, aph(3')-XV* | *tetG, catB, florR, sul1* |
| RPA185  (ST235) | *bla*_GES-5_ | *oprD*: del 1-899 | - | - | - | *mexZ*: del 293 (CGAGCCCGGTT) | F | C248T | T83I | C260T | S87L | *aac(6')-Ib-cr, aadA-6, aph(3')-XV* | *tetG, catB, florR, sul1* |
| RPA206  (ST235) | *bla*_GES-5_ | *oprD*: del 1-899 | - | - | - | *mexZ*: del 293 (CGAGCCCGGTT) | F | C248T | T83I | C260T | S87L | *aac(6')-Ib-cr, aadA-6, aph(3')-XV* | *tetG, catB, florR, sul1* |
| RPA23  (ST235) | *bla*_GES-5_ | *oprD*: del 1-899 | - | - | - | *mexZ*: del 293 (CGAGCCCGGTT) | F | C248T | T83I | C260T | S87L | *aac(6')-Ib-cr, aadA-6, aph(3')-XV* | *tetG, catB, florR, sul1* |
| RPA32  (ST235) | *bla*_GES-5_ | *oprD*: del 1-899 | - | - | - | *mexZ*: del 293 (CGAGCCCGGTT) | F | C248T | T83I | C260T | S87L | *aac(6')-Ib-cr, aadA-6, aph(3')-XV* | *tetG, catB, florR, sul1* |
| RPA41  (ST235) | *bla*_GES-5_ | *oprD*: del 1-899 | - | - | - | *mexZ*: del 293 (CGAGCCCGGTT) | F | C248T | T83I | C260T | S87L | *aac(6')-Ib-cr, aadA-6, aph(3')-XV* | *tetG, catB, florR, sul1* |
| RPA66  (ST235) | *bla*_VEB-16_ | *oprD*: C1270T | 424* | - | - | - | - | C248T | T83I | C260T | S87L | *aac(6')-I, aac(3)-Ic, aadA-6* | *catB, cml, sul1* |
| RPA91  (ST235) | *bla*_GES-15_ | *oprD*: del 1-899 | - | - | - | *mexZ*: del 293 (CGAGCCCGGTT) | F | C248T | T83I | C260T | S87L | *aac(6')-Ib-cr, aadA-6, aph(3')-XV* | *tetG, catB, florR, sul1* |
| JPAB50  (ST244) | *bla*_OXA-232_ | - | - | - | - | *mexZ*: del 293 (CGAGCCCGGTT) | F | - | - | - | - |  | *catB* |
| JPAU54  (ST308) | - | *oprD*: del 178 (G) | 94* | *nalD*: del 398 (GT) | 100* | - | - | C248T | T83I | C260T | S87L | *aac(3)-Id, aac(6')-I, aadA11,strAB* | *dfrB5, catB, floR, sul1, crpP, qnrVC3* |
| DPA57#  (ST357) | *bla*_VIM-2_ *bla*_OXA-10_ *bla*_VEB-9_ | *oprD*: del 300 (CCGACAAGAC) | 107* | *nalD*: del 398 (GT) | F | - | - | C248T | T83I | C260T | S87L | *aac(3)-Id ,aac(6')-I,aadA24, ant(2'')-Ia* | *dfrB2, dfrB5, tetA, catB, sul1, crpP, qnrVC3* |
| JPAB41  (ST357) | *bla*_OXA-10_ *bla*_VEB-9_ | *oprD*: Ins 233 (T) | 169* | *nalD*: del 398 (GT) | F | - | - | C248T | T83I | C260T | S87L | *aac(6')-I, aadA-1b, ant(2'')-Ia* | *dfrB2, tetA, catB, sul1, crpP* |
| RPA135#  (ST357) | *bla*_VIM-2_ *bla*_OXA-10_ *bla*_VEB-9_ | *oprD*: del 300 (CCGACAAGAC) | 107* | *nalD*: del 398 (GT) | F | - | - | C248T | T83I | C260T | S87L | *aac(3)-Id, aac(6')-I, aadA24, ant(2'')-Ia* | *dfrB2, dfrB5, tetA, catB, sul1, crpP, qnrVC3* |
| RPA226  (ST357) | *bla*_OXA-10_ *bla*_VEB-9_ | *orpD*: del 92 (C ) | 54* | *nalD*: del 398 (GT) | F | - | - | C248T | T83I | C260T | S87L | *aac(6')-I, aadA24, ant(2'')-Ia* | *dfrB2, tetA, catB, sul1, crpP* |
| RPA37  (ST357) | - | *oprD*: Ins 596 (A) | 199* | - | - | - | - | - | - | - | - | *-* | *catB, crpP* |
| DPA32  (ST500) | - | *oprD*: del 304 (A) | 110* | - | - | - | - | - | - | - | - | *-* | *catB, crpP* |
| MPA91#  (ST773) | *bla*_NDM-1_ | *oprD*: Ins 669 (CCTCAGCG) | 241* | - | - | - | - | C248T | T83I | C260T | S87L | *rmtB, aadA11* | *tetG, catB, sul1, qnrVC3* |
| JPAB38  (ST829) | - | *oprD*: G831A | 277* | - | - | - | - | A247G | T83A | - | - | *-* | *catB, crpP* |
| JPAR102  (ST865) | - | *oprD*: del 1181 (A) | 432* | *nalC*: C484T | 162* | - | - | - | - | - | - | *-* | *catB, crpP* |
| JPAO31  (ST1020) | - | - | - | - | - | - | - | - | - | - | - | *-* | *catB* |
| RPA100  (ST1076) | - | *oprD*: Ins 212 (C) | 74* | *mexR*: del 366 (GC) | 144* | - | - | - | - | - | - | *-* | *catB* |
| RPA117  (ST1659) | - | *oprD*: del 197 (CCCAAGGCTTC) | 70* | - | - | - | - | - | - | - | - | *-* | *catB,crpP* |
| JPAB24  (ST2374) | - | - | - | - | - | - | - | G259T | D87Y | - | - | *-* | *catB* |
| JPAB21  (SLV1567) | - | *oprD*: del 210 (CACCACCTAT) | 91* | *nalD*: del 516 (CCTGTTCAACGACTGGTTGCGCGAC) | F | - | - | - | - | - | - | *-* | *catB* |
| JPAU51  (ST1659) | - | *oprD*: del 52 (ACTCAGTTCGCCG) | 90* | - | - | - | - | - | - | - | - | *ant(2'')-Ia* | *catB* |
| MPA14  (ST235) | *bla*_GES-5_ | - | - | - | - | *mexZ*: del 293 (CGAGCCCGGTT) | F | C248T | T83I | C260T | S87L | *aac(6')-Ib-cr, aadA-6, aph(3')-XV* | *tetG, catB, florR, sul1* |
| JPAU94  (ST235) | *bla*_GES-5_ | *oprD*: del 1-899 | - | - | - | *mexZ*: del 293 (CGAGCCCGGTT) | F | C248T | T83I | C260T | S87L | *aac(6')-Ib-cr, aadA-6, aph(3')-XV* | *tetG, catB, florR, sul1, crpP* |
| HPA69  (ST500) | - | *oprD*: G526T | 176* | - | - | - | - | - | - | - | - | *-* | *catB, crpP* |
| RPA61  (ST1076) | - | *oprD*: IS 785 | - | - | - | - | - | - | - | - | - | - | *catB* |
| JPAU32  (ST375) | *bla*_VEB-9_ | *oprD*: del 1-1332 | - | *nalD*: del 398 (GT) | F | - | - | C248T | T83I | C260T | S87L | *aac(6')-I,ant(2'')-Ia* | *tetA, catB, sul1, crpP* |
| DPA39  (ST641) | - | - | - | - | - | - | - | - | - | - | - | - | *catB* |

(#) indicated the isolates that were also sequenced with the nanopore long-read technology. The first letter of each isolate name indicated the city of origin Al Ahsa (H); Al Madinah (M); Dammam (D); Jeddah (J) and Riyadh (R). Proteins labels indicated stop codons (*), frameshift (F) and truncated (T). (nuc) and (pro) in headers corresponded to nucleotide and protein.
